# A Pilot Study Investigating the Clinically Significant Association Between Overweight, Hyperlipidemia, and Insulin Resistance in Dogs With Chronic Liver Disease

**DOI:** 10.1155/vmi/4827011

**Published:** 2026-06-07

**Authors:** Aurora Cogozzo, Verena Habermaass, Rebecca Dini, Ilaria Lippi, Anna Pasquini, Alessio Pierini, Veronica Marchetti

**Affiliations:** ^1^ Department of Veterinary Sciences, University of Pisa, Pisa, Italy, unipi.it; ^2^ Centro Veterinario Pisani-Carli-Chiodo, Luni Mare, Italy

**Keywords:** canine liver disease, cholesterol, chronic hepatopathy, HOMA-IR, insulin resistance, metabolic, obesity, steatosis, triglycerides

## Abstract

The liver plays a central role in glucose and lipid metabolism. In human medicine, glucose metabolism alterations and insulin resistance are commonly associated with chronic liver disease (CLD), particularly in metabolic dysfunction–associated steatotic liver disease (MASLD). In veterinary medicine, however, the role of glucose metabolism in CLD remains poorly understood. We enrolled 67 client‐owned dogs, including healthy controls, hepatopathic dogs with (metabolic hepatopathy [MH]) or without (non‐metabolic hepatopathy [non‐MH]) metabolic factors such as overweight and hyperlipidemia, chronic kidney disease (CKD) dogs, and hyperlipidemic CKD (CKD HL) dogs. We measured serum liver enzymes, triglycerides, cholesterol, glucose, fructosamine, and insulin. Insulin resistance was assessed using the homeostasis model assessment for insulin resistance index (HOMA‐IR). Our findings revealed significantly elevated HOMA‐IR values in MH dogs compared to all other groups (median: MH—6.89; non‐MH—3.71; CKD—2.19; CKD HL—1.36; healthy—3.20). This increase occurred despite no significant differences in serum glucose and fructosamine concentrations. Results suggest the presence of insulin resistance in dogs with MH. HOMA‐IR was not correlated with liver biochemical parameters (ALT, ALP, and bilirubin) but showed a positive correlation with hypertriglyceridemia. As this is a pilot study, further research is needed to clarify the role of insulin resistance in canine hepatopathies.

## 1. Introduction

The liver plays a pivotal role in glucose and lipid metabolism through several pathways interacting with adipose tissue, gut, skeletal muscles, and kidneys [1]. Any disruption in this complex system can lead to the development of insulin resistance (IR), which is defined as “the presence of varying degrees of interference of insulin action on target cells” [2].

In human medicine, disorders of glucose metabolism and IR are commonly associated with chronic liver disease (CLD). CLD refers to a spectrum of chronic conditions characterized by hepatocellular injury, inflammation, and alterations in hepatic circulation and biliary function. These disorders are progressive in nature and may ultimately lead to fibrosis, cirrhosis, and liver failure [3, 4]. The underlying mechanisms of glucose dysmetabolism and IR vary among the different forms of CLD [5–7]. For example, in chronic viral hepatitis, the hepatitis C virus (HCV) can promote steatosis by its protein disturbing insulin signaling pathway. While there is some evidence linking hepatitis B virus (HBV) to IR, the underlying mechanisms remain unclear [8, 9]. Moreover, scientific evidence supports the presence of IR in chronic autoimmune liver diseases, such as autoimmune hepatitis (AIH), potentially driven by chronic inflammatory stimuli [10]. There is strong evidence supporting the association between IR and metabolic dysfunction–associated steatotic liver disease (MASLD), which is a pathological condition that affects 30% of the adult population worldwide [11]. MASLD is primarily defined by the accumulation of triglycerides within hepatocytes, with disturbances in glucose and lipid metabolism being the main drivers of steatotic liver disease [12]. Both altered glucid and lipidic metabolisms exacerbate each other and contribute to the progression of CLD [12]. IR reduces the inhibition of lipolysis, leading to increased serum free fatty acid concentrations. This contributes to the worsening of hepatic steatosis and the development of processes such as oxidative stress and lipotoxicity that can further promote IR [13, 14]. To date, MASLD is characterized by hepatocellular steatosis and metabolic syndrome, which is defined by the presence of at least one of the following criteria: elevated body mass index, high triglycerides, hyperglycemia, hypertension, and low HDL cholesterol [15, 16]. Both metabolic dysfunction and liver steatosis might contribute to the progression of MASLD toward metabolic dysfunction–associated steatohepatitis (MASH) and liver fibrosis, ultimately leading to cirrhosis [11, 15].

In veterinary medicine, the role of lipid metabolism in CLD remains poorly understood [17]. Hyperlipidemia is frequently observed in dogs with hepatopathies, often associated with cholestasis, endocrine disorders, dietary factors, or genetic predisposition [18–20]. A recent study showed a link between hepatocellular lipid accumulation and hepatic inflammation, associating hepatic inflammatory changes with conditions such as hyperlipidemia, obesity, biliary tract disease, and endocrinopathies [21].

Although IR in dogs has been documented in pathologic conditions such as Cushing’s syndrome, hypothyroidism, obesity, elevated progesterone levels, and other morbidities (i.e., inflammatory and traumatic status and pheochromocytoma) [22–25], to the best of the authors’ knowledge, no previous studies have evaluated IR in dogs with CLD.

The aim of this study was to assess the prevalence and magnitude of IR in dogs with CLD with and without metabolic factors such as hyperlipidemia and overweight. To achieve this aim, we compared dogs with CLD to healthy dogs and to dogs with chronic kidney disease (CKD), with and without hyperlipidemia.

We hypothesized that dogs with CLD and concurrent hyperlipidemia and/or overweight would exhibit IR, similarly to findings in human medicine. We also considered that, while hyperlipidemia may contribute to IR, CLD itself could play a significant role in the development of this condition.

## 2. Materials and Methods

This retrospective study was carried out in accordance with the Declaration of Helsinki and received approval from the Ethics Committee of the University of Pisa (protocol code no. 04/2025, approval date: 21/01/2025).

We enrolled client‐owned dogs referred to the Internal Medicine Service of the Veterinary Teaching Hospital of the University of Pisa between January 2024 and June 2025 with a diagnosis of CLD.

The diagnosis of CLD was based on a persistent (> 2 months) increase of two or more liver enzymes between alkaline phosphatase (ALP) > 250 U/L (reference range [RR]: 45–250 U/L), gamma‐glutamyl transferase (GGT) > 11 U/L (RR: 2–11 U/L), alanine aminotransferase (ALT) > 70 U/I (RR: 20–70 U/L), aspartate aminotransferase (AST) > 40 U/L (RR: 15–40 U/L), and concurrent hepatobiliary ultrasound alteration. Identified ultrasound abnormalities consisted of diffuse hyperechoic parenchyma, alterations in liver size and/or margins, presence of hepatic nodules suggestive of benign hyperplastic changes, abnormalities of the gallbladder wall, characterized by thickening and increased echogenicity, as well as alterations in its contents (such as mucoceles, biliary sludge not influenced by gravity, and cholelithiasis), chronic dilatation of intrahepatic bile ducts or the common bile duct, and mineral deposits within the intrahepatic biliary system [26].

Among dogs diagnosed with CLD, we identified two groups: those presenting with metabolic factors—specifically hyperlipidemia and/or overweight—were classified as having metabolic hepatopathy (MH), while those without such evidence were classified as non‐metabolic hepatopathy (non‐MH). Hyperlipidemia was defined as a 12‐h fasting serum cholesterol concentration > 280 mg/dL (RR: 120–280 mg/dL) and/or triglyceride concentration > 90 mg/dL (RR: 25–90 mg/dL). Overweight was defined as a body condition score (BCS) greater than 5 on a 9‐point scale.

Exclusion criteria included diabetes, hypothyroidism, hyperadrenocorticism, hepatic neoplasia, and vascular disorders of the liver (e.g., portosystemic shunt and portal hypoplasia) based on medical records, laboratory results, and imaging diagnostics. According to these criteria, dogs with significant comorbidities (e.g., cardiac, oncologic, hematologic, or infectious diseases) were also excluded.

Three control groups were included: healthy blood donors, hyperlipidemic nephropathic dogs (CKD HL), and nephropathic dogs without hyperlipidemia (CKD). Healthy dogs must show normal body weight, no evidence of hyperlipidemia, and unremarkable physical examination and hematobiochemical analysis. Dogs were classified as nephropathic (CKD) if they had a serum creatinine concentration greater than 1.5 mg/dL (RR: 0.6–1.5 mg/dL) and a confirmed diagnosis of CKD based on historical, clinical, ultrasonographic, and biochemical criteria. To meet inclusion criteria, CKD HL dogs must not be overweight, despite their hyperlipidemia status. Dogs with CKD and concurrent liver disease (defined as an increase in two or more liver enzymes accompanied by hepatobiliary abnormalities on ultrasound) were excluded. Dogs with nephrotic syndrome were also excluded; this was defined by the presence of proteinuria (persistent UPC ratio > 0.3), hypoalbuminemia (albumin < 2.6 g/dL), hypercholesterolemia (cholesterol > 280 mg/dL), and peripheral edema or ascites.

Serum liver enzymes, total bilirubin, triglycerides, cholesterol, glucose, fructosamine, total protein, albumin, creatinine, and urea were evaluated. Exceeding frozen serum from the included patients was stored at −80°C for no longer than 6 months and was analyzed with SAT‐450 biochemical analyzer (Assel S.r.l., Rome, Italy). Data on insulin stability in canine serum stored at −80°C are lacking; however, studies in cats (stable up to 15 months) [27] and humans (stable for over 5 years) [28] suggest good long‐term stability at this temperature.

Serum concentrations of insulin were measured using commercially available canine specific assays (Insulin ELISA kit, Mercodia, Uppsala, Sweden; code: 10‐1203‐01) [29]. IR was assessed by calculating the homeostasis model assessment for insulin resistance index (HOMA‐IR). HOMA‐IR was calculated with the linear approximation formulas ([glucose] × [insulin]/22.5) with glucose and insulin concentrations expressed in mmol/L and μU/mL, respectively [30, 31].

Statistical analysis was conducted using the software GraphPad Prism (Version 9, GraphPad Software, San Diego, CA, USA). Normal distributions of data were investigated through the Kolmogorov–Smirnov test. Continuous variables (age, HOMA‐IR, and biochemical parameters) were compared between groups with unpaired nonparametric tests (Kruskal–Wallis if > 2 groups and Mann–Whitney if 2 groups). Spearman correlation test was used to investigate correlations between continuous variables. Statistical significance was set at *p* < 0.05.

## 3. Results

### 3.1. Animals

Sixty‐seven dogs were enrolled in this retrospective case–control study. These were the patients who met the aforementioned inclusion criteria and had leftover serum samples available for analysis.

The study population comprised five groups of dogs, whose signalment and characteristics are presented in Table 1.

**TABLE 1:** Number of cases (N), sex distribution (female [F], male [M], neutered [N], and intact [I]), age (median year, range), breed, and group‐specific characteristics for each study group.

Group	*n*	Sex	Age, median (range)	Breed distribution	Group‐specific characteristics
MH	13	9 F (69%, 7 N, 2 I), 4 M (31%, 3 N, 1 I)	9 years (7–12)	7 (54%) mixed breed 2 (14%) Bernese Mountain Dogs 1 (8%) of each: Labrador Retriever, Pinscher, Miniature Pinscher, Australian Shepherd	9 (70%) hyperlipidemic 2 (15%) overweight 2 (15%) overweight and hyperlipidemic

Non‐MH	14	8 F (57%, 3 N, 5 I), 6 M (43%, 2 N, 4 I).	9 years (3–14)	4 (30%) mixed breed 2 (14%) Maltese 1 (7%) of each: French Bulldog, Jack Russell Terrier, Toy Poodle, Cocker Spaniel, Labrador Retriever, Bernese Mountain Dog, Setter, Chihuahua.	—

CKD	10	3 F (30%, 3 N) 7 M (70%, 7 I).	9.5 years (4–15)	2 (20%) mixed breed 2 (20%) Cavalier King Charles Spaniels 2 (20%) Golden Retrievers 1 (10%) of each: Bichon Frisé, Chihuahua, German Shepherd, Hound	3 IRIS stage 2 1 IRIS stage 3 6 IRIS stage 4

CKD‐HL	10	7 F (70%, 2 N, 5 I), 3 M (30%, 1 N, 2 I).	5.5 years (2–14)	2 (20%) Golden Retrievers 1 (10%) dog of each: mixed breed, Whippet, German Shepherd, Labrador Retriever, Dalmatian, American Staffordshire Terrier, Lagotto Romagnolo, Husky	3 IRIS stage 2 1 IRIS stage 3 6 IRIS stage 4

Healthy	20	9 F (45%, 4 N, 5 I), 11 M (55%, 5 N, 6 I).	6 years (3–8)	7 (35%) mixed breed 5 (25%) Labrador Retriever 2 (10%) Weimaraner 1 (5%) of each: Maremma Sheepdog, Cane Corso, Boxer, Bernese Mountain Dog, Greyhound, Golden Retriever.	—

Sex was evenly distributed across groups (*p* = 0.26). Age, however, differed significantly (*p* = 0.0005), with healthy controls and CKD HL patients being younger than the other groups.

Descriptive statistics using median values and their respective ranges of serum biochemical parameters for each group are presented in Table 2. Cholesterol, triglycerides, and glucose metabolism indicators—including glucose, fructosamine, and the calculated HOMA‐IR—are summarized using median values and their respective ranges in Table 3.

**TABLE 2:** Serum concentrations of hepatic enzymes, total bilirubin (Tot Bil), total protein (TP), albumin (Alb), creatinine (Crea), and urea (BUN) in each of the five groups.

Biochemical parameter	Healthy (*n* = 20)	CKD (*n* = 10)	CKD HL (*n* = 10)	Non‐MH (*n* = 14)	MH (*n* = 13)	Reference range
ALP (U/L)	77.5 (27–143)	108 (29–286)	158 (49–305)	393.5 (64–4871)	396 (96–2440)	45–250
GGT (U/L)	2.8 (0.9–7.1)	2.6 (0.4–6.8)	2.2 (0.6–4.4)	5.15 (0.4–208)	5.6 (1.6–62.8)	2–11
AST (U/L)	—	29 (18–45)	29.5 (16–50)	64 (15–493)	49 (19–113)	15–40
ALT (U/L)	47 (19–103)	57 (26–137)	44 (4.9–96)	152.5 (34–854)	246 (47–201)	20–70
Tot Bil (mg/dL)	0.22 (0.09–0.3)	0.165 (0.08–0.29)	0.155 (0.06–0.3)	0.19 (0.11–2.29)	0.21 (0.11–0.51)	0.07–0.3
TP (g/dL)	6.4 (5–7.6)	59 (4.9–7.7)	6.4 (4–8.3)	6.6 (4.8–8)	6.8 (3.6–7.9)	5.8–7.8
Alb (g/dL)	3.65 (2.1–4.3)	3.35 (2.2–3.9)	3.2 (2.3–4)	3.3 (2.4–4.4)	3.6 (2.5–4.8)	2.6–4.1
Crea (mg/dL)	1.2 (0.5–1.4)	5.9 (1.7–11.9)	7.25 (1.6–10.9)	1 (0.6–1.5)	0.8 (0.4–1.3)	0.6–1.5
BUN (mg/dL)	28 (14–54)	177.5 (68–394)	229 (41–341)	31 (15–69)	34 (19–40)	15–55

*Note:* Serum AST concentrations of healthy dogs are not reported because they are not included in the routinary blood donor profile.

**TABLE 3:** Serum concentrations of cholesterol (Chol), triglycerides (Trig), glucose (Glu), fructosamine (Fru), insulin, and HOMA‐IR in each of the five groups.

Biochemical parameter	Healthy (*n* = 20)	CKD (*n* = 10)	CKD HL (*n* = 10)	Non‐MH (*n* = 14)	MH (*n* = 13)	Reference range
Chol (mg/dL)	256 (83–277)	223.5 (53–263)	337.5 (308–442)	223.5 (101–279)	302 (146–428)	120–280
Trig (mg/dL)	—	51.5 (26–234)	57 (34–137)	54 (25–63)	72 (31–235)	25–90
Glu (mmol/L)	5.91 (4.1–6.72)	5.88 (4.05–6.77)	5.94 (4.22–7.99)	5.47 (4.22–6.66)	5.72 (4.55–8.16)	4.44–6.94
Fru (mmol/L)	302.5 (227–383)	256 (204–347)	271.5 (166–342)	275.5 (151–390)	261 (170–407)	170–430
Insulin (μU/mL)	11.4 (0.9–26.5)	9.2 (2.6–21.8)	5.7 (3.3–20.1)	16 (1.7–28.3)	24.3 (6.8–34.3)	—
HOMA‐IR	3.20 (0–6.67)	2.19 (0.8–6.56)	1.36 (0.79–5.35)	3.71 (0.5–8.11)	6.89 (1.44–9.3)	—

*Note:* Serum triglyceride concentrations of healthy dogs are not reported because they are not included in the routinary blood donor profile. No reference ranges currently exist for canine HOMA‐IR and insulin.

### 3.2. Glucose Metabolism

Age was not significantly correlated with glucose (*p* = 0.06), fructosamine (*p* = 0.11), HOMA‐IR (*p* = 0.29), or insulin levels (*p* = 0.16).

**FIGURE 1:**
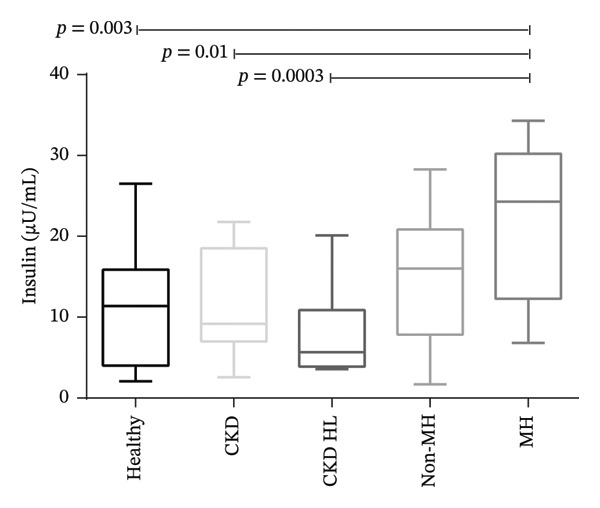
Serum insulin concentrations between the five study groups. Only statistically significant *p* values (*p* < 0.05) are shown. CKD = chronic kidney disease, CKD HL = chronic liver disease and hyperlipidemia, MH = metabolic hepatopathy, and non‐MH = non‐metabolic hepatopathy.

No statistically significant differences in glucose and fructosamine levels were observed among the groups (*p* = 0.81 and *p* = 0.41, respectively). A significant difference in insulin concentrations was found among the five groups (*p* = 0.004), as seen in Figure 1. In particular, among dogs with liver disease, whether associated with metabolic factors or not, median insulin levels appeared elevated. However, statistically significant differences in serum insulin concentrations were observed only between MH dogs and the healthy, CKD, and CKD HL groups.

**FIGURE 2:**
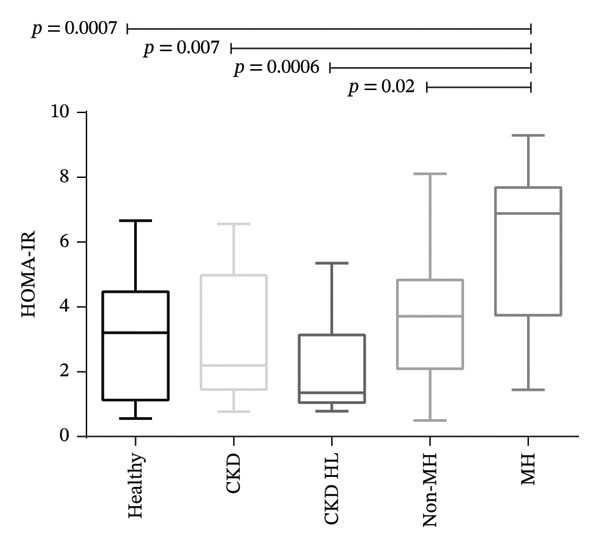
HOMA‐IR between the five groups of dogs. Only statistically significant *p* values (*p* < 0.05) are shown. CKD = chronic kidney disease, CKD HL = chronic liver disease and hyperlipidemia, MH = metabolic hepatopathy, and non‐MH = non‐metabolic hepatopathy.

Regarding HOMA‐IR, a statistically significant difference was observed among all study groups (MH, non‐MH, CKD, CKD HL, and healthy) (*p* = 0.004). Pairwise comparisons revealed that MH dogs exhibited significantly higher IR compared to all other groups, while no statistically significant differences were found among the remaining groups, as shown in Figure 2.

**FIGURE 3:**
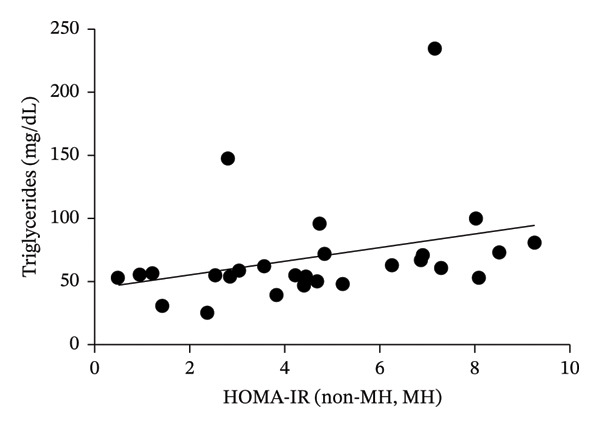
Correlation between triglycerides and HOMA‐IR in non‐MH and MH groups. MH = metabolic hepatopathy; non‐MH = non‐metabolic hepatopathy.

The investigation of a possible correlation between HOMA‐IR in hepatopathic patients (non‐MH and MH) and levels of cholesterol and triglycerides was performed. A moderate positive significant correlation was found only between HOMA‐IR and triglyceride levels (*p* = 0.013, *r* = 0.47, see Figure 3). No significant correlation was observed between HOMA‐IR and serum cholesterol concentration (*p* = 0.21). Similarly, no significant correlations were identified between HOMA‐IR and liver parameters, including ALP (*p* = 0.50), ALT (*p* = 0.08), and bilirubin (*p* = 0.24). Moreover, no statistically significant correlations were found between ALP, ALT, bilirubin, and serum lipid concentrations (cholesterol and triglycerides) (*p* > 0.05).

## 4. Discussion

This is a preliminary data study, the first to evaluate the presence of IR in canine patients with CLD.

In human medicine, IR is closely associated with CLD, in particular with MASLD phenotype. Therefore, it is linked to predisposing factors such as elevated body mass index and hypertriglyceridemia. In these patients, the HOMA‐IR index is used to clarify metabolic dysfunction in adults [11], and it has been demonstrated that there is a strict relationship between IR, lipid dysmetabolism, and steatosis [13, 14].

In dogs, IR has been observed in association with several conditions, including obesity and hormonally driven alterations (i.e., involving glucocorticoids or progesterone) [22]. However, an association between IR and CLDs has not been reported.

From the outset, it is important to clarify that a direct parallel with MASLD is not possible, as specific studies and diagnostic criteria are well established in human medicine but not yet available in veterinary medicine. Additionally, metabolic differences such as bile acid metabolism between humans and dogs could result in different clinical presentations.

While an overall elevation in median serum insulin levels was observed in all CLD dogs (MH + non‐MH groups), only the insulin levels in the MH group were significantly higher than those measured in the healthy, CKD, and CKD HL groups. This may reflect the central role of the liver in glucose and lipid metabolism, suggesting that it could contribute to IR even in the absence of metabolic factors. However, hyperlipidemia and overweight may further exacerbate IR, as evidenced by the significant differences observed between MH dogs and the other groups.

The findings for insulin levels are consistent with the HOMA‐IR results. In fact, the MH group showed an increased IR compared to the other groups (non‐MH, healthy, CKD, and CKD HL). Interestingly, no significant differences in HOMA‐IR were observed among these latter groups.

Despite the increase in the HOMA‐IR index, our study did not reveal significant differences in serum glucose and fructosamine concentrations—parameters commonly used in clinical practice to assess glucose metabolism—between the MH and non‐MH groups. This suggests caution in excluding alterations in glucose metabolism based solely on normal glucose and fructosamine values.

In our cohort of canine patients with MH, only two were classified as obese, two presented both obesity and hyperlipidemia, and nine showed hyperlipidemia alone. Based on these findings, we hypothesize that hyperlipidemia may represent a concurrent factor in the pathogenesis of IR—similarly to what has been described in human medicine.

However, no statistical difference was found between HOMA‐IR in CKD HL group and the other groups, suggesting that hyperlipidemia alone may not be the only driving factor for IR occurrence. Based on this, it can be hypothesized that hyperlipidemia could act as a concurrent factor for IR primarily when associated with liver disease. This assumption may be supported by central role of the liver in carbohydrate and lipid metabolism, a function that is not equivalently performed by the kidney. In fact, the liver is deeply involved in these metabolic processes as it is responsible for glycogenolysis, glycogenesis, and gluconeogenesis, as well as the synthesis and oxidation of fatty acids. Although the kidney also participates to a lesser extent in some of these metabolic pathways, such as gluconeogenesis, it does not have the same capacity as the liver to coordinate carbohydrate and lipid metabolism [32].

Although the difference in HOMA‐IR between the CKD group and the other groups did not show statistical significance, it is interesting that the median HOMA‐IR value tended to be lower in CKD and CKD HL patients compared to other groups. This finding is potentially consistent with a recent study conducted on nondiabetic feline patients with IRIS stage 3‐4 CKD [33], which reported a reduction of the HOMA‐IR index compared to healthy controls, suggesting a possible dysfunction of pancreatic beta cells due to increased production of reactive oxygen species (ROS) [34, 35]. Despite interspecies differences, similar trends were observed in both canine and feline patients. This contrasts with the human medical literature that frequently reports a high HOMA‐IR associated with CKD patients [36].

Regarding the correlation between the HOMA‐IR index and cholesterol and triglyceride concentrations, only the latter showed a moderate positive significant correlation. This finding may be explained by the strict relation between triglycerides and hepatocellular steatosis. In fact, hepatocellular steatosis consists in an intracellular accumulation of lipids, primarily triglycerides [37]. As reported in human medicine, serum hypertriglyceridemia is associated with hepatic steatosis secondary to IR [38]. Similarly, a comparable mechanism may be hypothesized in canine patients, whereby elevated serum triglycerides contribute to worsening IR and potentially promote steatotic changes.

Moreover, in human metabolic syndrome associated with MASLD, hypertriglyceridemia is one of the diagnostic criteria. As for cholesterol, a distinction is made between different lipoprotein fractions, with metabolic syndrome being associated specifically with decreased HDL cholesterol levels. In our study, we measured total cholesterol without differentiating between HDL and LDL fractions. This may represent a possible explanation for the lack of correlation observed between total cholesterol and the HOMA‐IR index, whereas a statistically significant positive correlation was found with triglyceride levels. In this context, a comprehensive lipid characterization, including a lipidogram and measurement of HDL/LDL fractions, would strengthen the interpretation of our findings.

No significant correlation was observed between liver enzymes (ALP, ALT, and bilirubin) and the HOMA‐IR index. This lack of association may be due to the fact that serum liver enzymes, mostly indicating cytolytic injury, in dogs with concurrent hepatopathy may not consistently reflect the severity and extent of the CLD itself [39]. In particular, there is evidence of canine chronic hepatitis confirmed by histology in the absence of increased serum liver enzyme activity [40–43]. Another important consideration is that in late‐stage cirrhosis, transaminase activity may actually decrease due to parenchymal loss [44–46]. Even in human medicine, a nonlinear association between liver enzyme levels and the presence and progression of MASLD has been reported, which limits the early diagnosis of the disease [11]. Furthermore, it should be considered that IR may follow a different pathogenetic pathway and thus occur independently of liver cytolytic injury itself. Another hypothesis to consider is that IR could even precede any detectable increase in serum markers of liver damage. Similarly, serum total bilirubin mostly represents a marker of reduced function or cholestasis [45]; therefore, it is potentially not directly related to metabolic alterations such as IR.

The absence of a significant correlation between hyperlipidemia and serum markers of liver injury (ALT, ALP, and bilirubin) may reflect the complexity of the disease. In human MASLD, steatosis may often occur in the absence of biochemical alterations. Hepatocellular injury markers may be rarely elevated, often remaining within normal ranges despite the presence of hyperlipidemia, which represents a current challenge for the assessment of the severity of steatosis [11]. Regarding our study, it is important to first acknowledge that the limited sample size may have influenced the results. Additionally, it is worth considering that, among dogs with hyperlipidemia, steatosis might develop only in a subset of cases, and elevated liver enzyme levels could occur in only a fraction of those, if at all.

The main limitation of this study is the absence of histopathological evaluation in dogs with CLD, which would have allowed a more precise grading of hepatic inflammation and fibrosis. Furthermore, the small sample size represents a major limitation of the study, particularly considering the division of the population into five groups. The lack of age homogeneity among groups is another limitation, as the healthy and CKD HL groups were significantly younger than the others. Lastly, the lack of data on adiposity distribution and the absence of additional parameters for a more comprehensive assessment of glucose metabolism (such as glycated hemoglobin, HbA1c) should also be acknowledged.

An interesting future perspective would be to investigate histological, histochemical, and immunohistochemical alterations, considered more precise and reliable indicators of disease status in chronic hepatopathic patients, in correlation with disturbances in glucose and lipid metabolism and the HOMA‐IR index. This would allow for a better characterization of the clinical picture and a deeper understanding of the pathogenetic mechanisms underlying IR in CLD, thereby more accurately evaluating the contribution of potential metabolic factors. Another interesting direction for future research could be a longitudinal study monitoring IR in chronic hepatopathic canine patients over time. This could include the evaluation of HOMA‐IR, glucose tolerance tests, and the correlation of these parameters with disease progression. Such a study would help us not only better understand the underlying mechanisms linking IR and chronic hepatopathy but also explore whether IR may serve as a prognostic marker.

## 5. Conclusions

Our study suggests the possibility of IR in patients with CLD and concurrent metabolic factors (hyperlipidemia and/or overweight), as evidenced by the HOMA‐IR index, despite normal serum glucose and fructosamine. This metabolic alteration may play an important role in the progression of CLD, as reported in human medicine. At this stage, the clinical significance of this finding is not conclusive. Given the pilot nature of the study, larger and more in‐depth investigations are warranted to better characterize these patients, evaluate their clinical progression, and clarify the underlying pathogenic mechanisms, including the role of IR in hepatopathic dogs.

**Nomenclature**
AIH: Autoimmune hepatitis
Alb: Albumin
ALP: Alkaline phosphatase
ALT: Alanine aminotransferase
AST: Aspartate aminotransferase
BCS: Body condition score
BUN: Urea
Chol: Cholesterol
CKD: Chronic kidney disease
CKD HL: Chronic kidney disease with hyperlipidemia
CLD: Chronic liver disease
Crea: Creatinine
Fru: Fructosamine
GGT: Gamma‐glutamyl transferase
Glu: Glucose
HbA1c: Glycated hemoglobin
HBV: Hepatitis B virus
HCV: Hepatitis C virus
HOMA‐IR: Homeostasis model assessment for insulin resistance index
IR: Insulin resistance
MASLD: Metabolic dysfunction–associated steatotic liver disease
MASH: Metabolic dysfunction–associated steatohepatitis
MH: Metabolic hepatopathy
Non‐MH: Non‐metabolic hepatopathy
RR: Reference range
Tot Bil: Total bilirubin
TP: Total protein
Trig: Triglycerides
UPC ratio: Urine protein creatinine ratio

## Funding

The present study received no external funding.

Open access publishing facilitated by Universita degli Studi di Pisa, as part of the Wiley ‐ CRUI‐CARE agreement.

## Conflicts of Interest

The authors declare no conflicts of interest.

## Data Availability

The data that support the findings of this study are available from the corresponding author upon reasonable request.
